# Crumbled or mashed feed had no significant effect on the performance of lactating sows or their offspring

**DOI:** 10.1186/s40781-015-0078-9

**Published:** 2015-12-23

**Authors:** S. C. Kim, H. L. Li, J. H. Park, I. H. Kim

**Affiliations:** Department of Animal Resource and Science, Dankook University, Cheonan, Choongnam 330-714 South Korea

**Keywords:** Feed processing, Performance, Piglets, Sow

## Abstract

**Background:**

Physical and chemical properties of feedstuffs can be changed by feed processing. Moreover, through various mechanisms, feed processing can affect growth performance and feed efficiency of swine, nutrition value of the feed. Weaning-to service-intervals (WSI), subsequent farrowing rates, and total-born litter sizes were determined by feed intake and metabolic state during lactation.

**Methods:**

A total of 20 sows (Landrace × Yorkshire) with an average body weight (BW) of 266.1 kg 4 d before farrowing were used to determine the effect of feed processing on the performance of lactating sows and their offspring. The following two dietary treatments were used: 1) Crumble diet (C); and 2) Mash diet (M). Ten replications were used for each treatment. Back fat thickness of sows was measured 6 cm off the midline at the 10th rib using a real-time ultrasound instrument at 4 d before farrowing, 1 d after farrowing, and during weaning. Sow BW were also checked at 4 d before farrowing, 1 d after farrowing, and during weaning. Fecal score of sows were assessed on d 14. Fecal score of piglets were observed on d 7, 15, and 24. Data were analyzed using *t*-test procedure of SAS (2014) with sow as experimental unit.

**Results:**

No significant (*p* > 0.05) difference was observed in the reproduction performance of sows between the two treatments. In addition, there was no significant (*p* > 0.05) difference in the growth performance of piglets between the two treatments. Fecal score of sows or piglets showed no significant (*p* > 0.05) difference either.

**Conclusions:**

In conclusion, different feed processing (mash or crumble) did not make any significant difference on the performance of lactation sow or their piglets.

## Background

Feed intake and metabolic state during lactation can influence WSI, subsequent farrowing rates, and total-born litter sizes [1]. Therefore, insufficient feed intake of sows during lactation is a serious problem because sows require large amount of energy for high milk production during lactation. A low feed intake during lactation can lead to great body weight (BW) loss, decreased milk production, and reproductive problems that may lead to culling of the sows [2, 3]. However, genetic selection for large litter size can lead to heavy burden of milk production [4].

Feed processing can change the physical and chemical properties of feedstuffs. In addition, it can also improve the nutrition value of the feed through various mechanisms. Feed processing of diets has been extensively used in commercial feed production. Compared to mashed diet, expanded crumble diet can decrease feed cost per kg weight gain by 15 % [5]. It is known that pelleting feed can improve the growth performance and feed efficiency in swine [6–9]. However, to our knowledge, few studies have evaluated the use of crumble diets and mashed diets in lactating sows. Therefore, the objective of this study was to evaluate the effect of crumbled and mashed diets on the performance of lactation sows and their offspring.

## Methods

The experimental protocols describing the management and care of animals were reviewed and approved by the Animal Care and Use Committee of Dankook University.

### Experimental design, animals, housing and diets

A total of 20 sows (Landrace × Yorkshire) with an average BW of 266.1 kg (measured 4 days before farrowing) were used in this experiment. Gestating sows were fed with basal gestation diet until farrowing. The diet was changed to experimental feed during the lactating period. On d 107 of gestation, sows were moved into farrowing crates in an environmentally regulated farrowing house and assigned to one of the following two dietary treatment groups: 1) Crumble diet (C), and 2) Mashed diet (M). Ten replications were used for each treatment. Diets were formulated to meet or exceed the NRC [10] nutrient requirements for sows (Table 1) . Farrowing crates (2.1 m × 0.6 m) included an area (2.1 m × 0.6 m) for newborn pigs on each side. Supplemental heat was provided for pigs using heat lamps (500 W) to keep the temperature constant at 35 °C for newborn piglets.

On the day of parturition, sows were not offered any feed. On the first day after farrowing, sows were fed with 1.5 kg lactation diet and 2.0 kg on the second day. The daily feed allowance was increased gradually by 0.5 kg per day. One week after farrowing, sows were provided experimental lactation feed and water *ad libitum*. All diets were provided in crumbled form or mashed form.

### Sampling and measurements

The back fat thickness of sows was measured 6 cm off the midline at the 10th rib using a real-time ultrasound instrument (Piglot 105, SFK Technology, Herlev, Denmark) 4 d before farrowing, 1 d after farrowing, and during weaning. The BW of sows were checked at 4 d before farrowing, 1 day after farrowing, and during weaning. Individual piglet BW was assessed on d 0 (birth weight), 7, 14, 21, and 28 (weanling). The number of piglets for every sow was recorded on the farrowing day and the weaning day to evaluate the survival rate of piglets. The number of birth piglet and live piglets were recorded on the farrowing day to calculate the rate of stillbirth. Feed intake of sows were recorded daily to determine the daily feed intake during lactation.

Detection of estrus was conducted twice per day from weaning onward at 08:30 and 16:00 daily. A sow was considered to be in estrus when it exhibited a standing response induced by a back pressure test when in the presence of a boar. Fecal score of sows was observed and recorded on d 14. The fecal score of piglets was observed and recorded on d 7, 15, and 24. To assess the fecal score, feces from each pig were scored by determining the moisture content according to the method described by Hu et al. [11]. Briefly, the following scoring system was used. Score of 1 indicated hard feces. Score of 2 was used for feces that were firm and well formed. Score of 3 indicated soft and partially formed feces. Score of 4 was for loose and semi-liquid feces. Score of 5 indicated watery feces.

### Statistical analysis

Data were analyzed using *t*-test procedure of SAS [12] with sow as experimental unit and farrowing group as block. The back fat thickness of sow and changes during lactation were analyzed using fat depth at farrowing as covariates. Piglet birth weight was used as a covariate for weaning weights during lactation. Lactation length was used as a covariate for the number of pigs weaned, survivability, weaning weights of sows and piglets, sow ADFI, and back fat change. Difference with *p* value level less than 0.05 was considered as statistically significant.

## Results

### Litter performance

The two dietary treatments had no significantly (*p* > 0.05) different effect on the reproduction performance of sows (Table 2).

**Table 1:** Sow diet composition (as-fed basis)

Items	Gestation diet	Lactation diet
Ingredients (%)		
Corn	57.10	51.12
Soybean meal (46 % CP)	10.65	24.61
Wheat bran	12.00	4.00
Rapeseed meal	3.70	2.50
Rice bran	6.00	5.00
Tallow	3.59	6.05
Molasses	3.60	3.50
Dicalcium phosphate	1.52	1.64
Limstone	0.99	0.76
Salt	0.60	0.50
L-lysine HCl (98 %)	0.05	0.12
Vitamin premix^a^	0.10	0.10
Mineral premix^b^	0.10	0.10
Calculated composition		
ME (MJ/kg)	3.19	3.44
CP (%)	13.10	17.10
Crude fat (%)	6.89	9.10
Lys (%)	0.65	1.00
Ca (%)	0.87	0.85
P (%)	0.76	0.73

^a^Provided per kilogram of complete diet: vitamin A, 10,000 IU; vitamin D3, 2000 IU; vitamin E, 48 IU; vitamin K3, 1.5 mg; riboflavin, 6 mg; niacin, 40 mg; d-pantothenic, 17 mg; biotin, 0.2 mg; folic acid, 2 mg; choline, 166 mg; vitamin B6, 2 mg; and vitamin B12, 28 μg

^b^Provided per kilogram of complete diet: Fe (as FeSO4⋅7H2O), 90 mg; Cu (as CuSO4⋅5H2O), 15 mg; Zn (as ZnSO4), 50 mg; Mn (as MnO2), 54 mg; I (as KI), 0.99 mg; and Se (as Na2SeO3⋅5H2O), 0.25 mg

**Table 2:** Effects of feed processing method on litter performance in lactation sows^a^

Items	C^b^	M^b^	*p*-value
Litter size, head			
Birth piglets No.	11.3 ± 0.5	11.7 ± 0.6	0.6307
Live piglets No.	10.4 ± 0.5	10.4 ± 0.6	1.0000
Weaning piglets No.	9.9 ± 0.4	9.8 ± 0.7	0.8973
Stillbirth rate^c^ (%)	7.61 ± 2.39	10.93 ± 3.78	0.4672
Survival rate^d^ (%)	95.19 ± 1.50	94.23 ± 2.33	0.6292
Body weight (kg)			
d 4 Before farrowing	263.7 ± 5.0	268.5 ± 5.1	0.5144
d 1 After farrowing	243.6 ± 5.0	246.8 ± 5.4	0.6637
Weaning (d 28)	236.1 ± 5.5	240.1 ± 5.6	0.6187
Body weight loss 1^e^	20.2 ± 0.6	21.7 ± 0.7	0.1112
Body weight loos 2^e^	7.5 ± 1.0	6.8 ± 1.1	0.6489
Backfat thickness (mm)			
d 4 Before farrowing	19.8 ± 0.5	19.9 ± 0.5	0.8932
d 1 After farrowing	19.5 ± 0.4	19.6 ± 0.5	0.8705
Weaning	15.3 ± 0.4	15.1 ± 0.4	0.7521
Backfat thickness loss 1^f^	0.3 ± 0.2	0.3 ± 0.2	1.0000
Backfat thickness loos 2^f^	4.2 ± 0.3	4.5 ± 0.3	0.4872
Average daily feed intake (g)	6.45 ± 0.01	6.45 ± 0.01	0.9784
Estrus interval (d)	5.3 ± 0.3	5.2 ± 0.2	0.7642

^a^Abbreviation: C; Crumble diet, M; Mash diet

^b^Mean ± Standard error

^c^Stillbirth rate : (Birth piglets No. – Live piglets No.) / Birth piglets No. × 100

^d^Survival rate : Weaning piglets No. / Live piglets No. × 100

^e^Body weight loss: 1, d 4 Before farrowing to d 1 After farrowing; 2, d 1 After farrowing to weaning

^f^Backfat thickness loss: 1, d 4 Before farrowing to d 1 After farrowing; 2, d 1 After farrowing to weaning

### Growth performance

The two dietary treatments had no significantly (*p* > 0.05) different effect on the growth performance of piglets (Table 3).

**Table 3:** Effects of feed processing method on growth performance in suckling piglets^a^

Items	C^b^	M^b^	*p*-value
Body weight (kg)			
Birth	1.317 ± 0.059	1.352 ± 0.041	0.6344
wk 1	2.601 ± 0.059	2.618 ± 0.053	0.8303
wk 2	4.293 ± 0.111	4.313 ± 0.082	0.8867
wk 3	6.221 ± 0.149	6.196 ± 0.114	0.8949
weaning (d 28)	7.687 ± 0.181	7.619 ± 0.167	0.7858
Average daily gain (g)			
wk 0 to 1	182 ± 4.2	179 ± 3.3	0.5714
wk 1 to 2	245 ± 7.5	238 ± 10.8	0.5918
wk 2 to 3	273 ± 5.2	264 ± 7.2	0.3215
wk 3 to weaning (d 28)	213 ± 11.3	203 ± 12.5	0.5924
Overall	230 ± 3.8	223 ± 6.9	0.3671

^b^Abbreviation: C; Crumble diet, M; Mash diet

^b^Mean ± Standard error

### Fecal score

The two dietary treatments had no significantly (*p* > 0.05) different effect on fecal scores of sows or piglets (Table 4).

**Table 4:** Effects of feed processing on fecal score in lactation sows and suckling piglets^a^

Items	C^b^	M^b^	*p*-value
Sow fecal score			
d 14	2.9 ± 0.1	2.8 ± 0.1	0.7142
Piglet fecal score			
d 7	3.3 ± 0.1	3.4 ± 0.1	0.3823
d 15	3.2 ± 0.1	3.2 ± 0.1	0.6601
d 24	3.0 ± 0.0	3.0 ± 0.0	0.9999

^a^Abbreviation: C; Crumble diet, M; Mash diet

^b^Mean ± Standard error

## Discussion

For the past several decades, nutritionists have attempted to further improve the nutritional value of swine feeds. Extruder and expander processing has been introduced. According to previous studies, the nutritional value of feeds may be altered by extruder/expander conditions such as the degree of cooking, preconditioning, and temperature [13, 14]. On the other hand, it is widely accepted that pelleting of diets could improve average daily gain and feed conversion ratio in pigs. Based on 16 trials, Ohh [15] has summarized that pelleted diet could improve the growth of swine by 3–4 % compared to mashed diet. In addition, it has been reported that pelleting could increase ADG while reducing pelleting and particle size could improve FCR [16].

Currently, much interest has been focused on the extruding/expanding technology for the manufacturing of swine feeds. In pig feed processing, expander processing method was introduced to improve pellet quality. Several researchers have reported that the expander processing has little effect on the performance of growing-finishing pigs fed on common diet [17]. There was no significant difference between mashed diet and heat-processed diet (pellet and expanded crumble) in improving ADG and ADFI. It has been previous reported that feeding pelleted diets could significantly increase FCR [5]. Increased nutrient digestibility and decreased feed wastage might have improved feed utilization in pigs feeding with pelleted diets [18]. Burnham et al. [19] have emphasized extrusion of soybean can improve rate and efficiency of gain when fed to nursery pigs in the place of soybean meal. While Kim t al. demonstrated that extrusion yielded a full-fat soy product had greater nutritional value than roasting [13]. of Furthermore, it has been reported that pelleting feed could improve gain/feed ratio by 7 % compared to mashed feed [20]. Many previous studies have also demonstrated that the efficiency of growth is improved when pigs are fed pelleted diets [17, 18, 21, 22]. Reducing diet particle size can improve the efficiency of growth in growing-finishing and nursery pigs [23, 24]. In addition, reduced diet particle size could increase digestibility of nutrients which may lead to improve the lactation performance in sows [25, 26]..

Wondra et al. [16] have reported that pelleting diet can increase ADG and gain/feed ratio by 5 and 7 %, respectively. Baird [27] has reported that pelleting corn-soybean meal diet can improve ADG and gain/feed ratio of growing pigs by 5 and 8 %, respectively. However, several experiments including the NCR-42 Committee on Swine Nutrition [28] failed to demonstrate that pelleting could consistently improve ADG. The mechanisms on how pelleted diets could increase ADG of pigs is currently unclear.

Several researchers have reported that the expander processing has little effect on the performance of growing-finishing pigs fed with common diets [17]. Previous studies have demonstrated that dressing percentage and back fat thickness are not affected by expander processing of pig diets [14, 29]. In addition, Yang et al. [5] have reported that expanding process may not be suitable for growing and finishing pigs because the expanding process will increase feed cost without improving the performance of pigs compared to the pelleting process. Our results showed that crumbled feed failed to significantly improve the litter performance compared to mashed feed. This might be due to the fact that the sows used in our study might have higher feed intake than sows used in other lactation experiments [30, 31]. Therefore, lactation sows might have obtained enough nutrients such as protein and energy regardless which type of feed processing was used.

## Conclusions

In conclusion, different feed processing did not make any significant difference on the performance or growth performance of lactation sows or their piglets.
